# Case of Popliteal Aneurism Cured by Compression over the Tumor

**Published:** 1851-09

**Authors:** H. N. Bennett

**Affiliations:** Bethel, Ct.


*Case of Popliteal Aneurism Cured by Compression over the Tumor.* By H. N. Bennett, M. D., of Bethel, Ct.—The following case possesses no intrinsic value, with the exception of the method of cure, which consisted in pressure applied directly over the aneurismal tumor, and it is for this reason only, that I have thought it worthy of record. Perhaps the fact of the patient having *cured himself* affords some additional interest, as I believe, in this respect, the cure is unique.

Thomas J. Elwell, a blacksmith, 30 years of age, during the winter of 1842–3, suffered from pain about the knee joint, which he supposed to be of a rheumatic character, and applied only domestic remedies for its relief. After a walk of twenty-two miles over frozen ground, the pain and lameness of the knee increased to such a degree, that he consulted me in reference to it. Upon examination, I immediately detected a pulsating tumor in the popliteal region, near the size of a hen's egg, and having all the characteristics of aneurism. This was in the month of March, 1843. At the end of five or six weeks from this date, the tumor had increased considerably in size, so much, that I advised my patient to submit to ligation of the femoral artery at once, as the nature of the difficulty could not be doubtful, and as I had at that time little confidence in any other mode of treatment. Feeling unwilling to endure a surgical operation, he soon went to New York for further advice. He consulted Drs. Mott and Rogers, who both gave him the same opinion which I had done, and urged him to remain at the New York Hospital for operation. He then passed over to Staten Island on a visit to his friends, where he consulted some physician, whose name I cannot recall, who also pronounced it a case of aneurism, but recommended him to try the effects of pressure upon the tumor. He returned home and commenced the application of pressure, *himself*, by bandaging the limb from the toes to the knee, and placing a compress of folded cloth directly upon the pulsating tumor. He persisted in this course for two or three months without the least benefit, when he determined to substitute a firmer compress. Instead of folds of cloth, folds of *sheet-lead* formed the nucleus of his compress, which was applied in the same manner as before. I called upon him occasionally to watch the progress of his treatment, and after the end of ten days after the application of the lead compress, I found him suffering much pain in the knee joint, from the severe pressure which he had made, but no diminution in the size of the tumor or the force of the pulsations, the latter being readily felt through the compresses when applied with all the force which he could endure. I advised him to discontinue what I considered a hazardous proceeding, and to abbreviate his sufferings by submitting to the usual operation. Notwithstanding, being a man of very strong resolution, he determined to pursue this course still longer, and abide the issue. At this time, however, he threw aside the lead compress, and filled its place with *a ball of caoutchouc*, which he bound upon the tumor with all the force he could endure. Not long after, perhaps two or three weeks, I called again to see him, and was much surprised upon examining the limb (which he exhibited with no little triumph,) to find the tumor much diminished in size, and the *pulsation gone.* I could also distinctly feel several arteries pulsating about the knee joint, evincing an increased development of the anastomosing branches of the femoral. From this time, the tumor in the popliteal space was gradually absorbed, and at the end of a few months had entirely disappeared. This man has since died of phthisis, in New York city.

I think there can be no question in this case, in reference to the diagnosis, since the most experienced surgeons in the country examined the patient, and had no hesitation in pronouncing it a case of aneurism, and recommending ligature of the femoral artery.—*New York Journal of Medicine, July*, 1851.

